# Segmental trans-endplate pedicle screws do not induce spinal deformity in a porcine model

**DOI:** 10.1007/s43390-026-01313-1

**Published:** 2026-03-30

**Authors:** Taylor R. Johnson, Talissa O. Generoso, Christine Farnsworth, Amishi Jobanputra, Jonathan H. Wen, Austin J. Stoner, Arianne Salunga, Vivian Ho, Miranda Guzman, David Berry, Vidyadhar Upasani, John S. Vorhies

**Affiliations:** 1 Department of Orthopaedic Surgery, Lucille Packard Children’s Hospital, School of Medicine, Stanford University, 453 Quarry Rd, 3Rd Floor, MC 5658, Stanford, CA USA; 2 Rady Children’s Hospital, University of California San Diego, San Diego, CA USA; 3 University of California San Diego, San Diego, CA USA

**Keywords:** Spinal growth modulation, Spinal deformities, Spinal epiphysiodesis trajectory (SET) screws, Trans-endplate screws

## Abstract

**Purpose:**

Growth modulation is an established technique for limb deformity correction and is increasingly applied to spinal deformities. While distraction-based posterior and anterior compressive methods have been explored, spinal growth modulation through fixation across vertebral growth centers remains unstudied. We hypothesized that unilateral trans-endplate screws—spinal epiphysiodesis trajectory (SET) screws—could induce partial anterior growth arrest and promote scoliotic deformity in a porcine model.

**Methods:**

Four male piglets (two experimental, two control) underwent unilateral posterior spinal instrumentation at four lower thoracic levels at eight weeks of age. Experimental animals received trans-endplate SET screws; controls received pedicle screws. Radiographs obtained three months postoperatively assessed vertebral height and Cobb angles. MRI and CT were also used to evaluate vertebral wedging, disc and facet health, physeal bars, and endplate changes.

**Results:**

After three months, no significant differences in coronal or sagittal Cobb angles were observed between SET and pedicle screw groups (*p* > 0.05). No vertebral wedging or restriction of vertical growth was seen in either group. Disc and facet health remained unchanged by Pfirrmann and Fujiwara grading. No physeal bars were identified; one SET specimen showed endplate irregularities.

**Conclusion:**

In this pilot porcine model, SET screws did not produce scoliotic or kyphotic deformity. Further research is necessary to clarify the mechanisms and timing required for effective anterior spinal growth modulation.

**Level of evidence:**

IV.

## Introduction

Growth modulation is a valuable tool in pediatric orthopedics, offering the potential to guide growth in the immature skeleton and correct deformities without the need for more invasive procedures. While growth modulation is commonly used to treat lower limb conditions, such as angular deformities and leg length discrepancy [1–5], its application is increasingly popular in the management of spinal deformity.

Spinal growth modulation via distraction-based posterior instrumentation [6–9] and anterior compressive methods [10] has been studied; however, mechanical fixation across vertebral growth centers remains largely unexplored. Anterior spinal growth modulation, as in the case of vertebral body tethering (VBT), allows gradual deformity correction without the need for spinal fusion [11, 12], but both thoracoscopic and open anterior approaches carry notable surgical risks [13, 14]. Posterior spinal approaches are more commonly used and familiar to spine surgeons; however, dependable methods for achieving anterior growth modulation from a posterior approach have not been developed [7].

The eccentric placement of trans-physeal screws is an effective technique for growth modulation through hemi-epiphysiodesis and has been used to gradually correct angular deformity in various settings [1–5]. In spinal surgery, the placement of trans-endplate screws has been shown to be anatomically feasible and safe. This can be achieved by introducing cranially directed pedicle screws that traverse the superior endplate of a vertebra and, in some cases, cross the intervertebral disc to engage the inferior endplate of the vertebra above. This technique, known as the pedicular transvertebral screw method, has demonstrated favorable outcomes in the setting of fusion for treatment of high-grade spondylolisthesis [7, 15, 16]. More recently, thoracic trans-discal and trans-endplate screw trajectories have shown promising biomechanical stability in cadaveric studies [18] and in vivo [18–20] suggesting their utility to enhance bone fixation. The potential for this screw trajectory to cause growth modulation has not been tested.

In this context, trans-endplate spinal epiphysiodesis trajectory (SET) screws, placed eccentrically into the superior vertebral endplate without crossing the disc, could provide a means of growth modulation for the anterior spine while preserving segmental biomechanical properties and intervertebral disc health. When placed bilaterally, a series of trans-endplate SET screws could symmetrically arrest anterior spinal growth, and when placed unilaterally, this technique could achieve partial anterior growth arrest, offering the potential to modulate spinal growth and correct pediatric spinal deformities.

To test this hypothesis, we propose that a series of trans-endplate SET screws placed unilaterally will induce partial anterior growth arrest sufficient to create scoliosis in an animal model. This pilot study aims to evaluate the potential of trans-endplate SET screws to affect anterior growth by comparing changes in coronal and sagittal curve magnitudes between preoperative and post-growth X-rays, assessing vertebral body and intervertebral disc wedging on post-growth Computed Tomography (CT) scans, and assessing intervertebral disc health using Magnetic Resonance Imaging (MRI).

## Materials and methods

This study was performed under an Institutional Animal Care and Use Committee (IACUC)-approved protocol. A porcine model was chosen given the rapid growth during the first four months of life and prior evidence that pedicle screws can induce spinal deformity in immature farm pigs [21]. Four immature pigs were divided into two groups: 1) two with placement of unilateral thoracic pedicle screws (on the right side of the vertebrae in one animal and left side in the other), and 2) two that underwent placement of unilateral SET screws (one on the right and one on the left side).

### Surgical protocol

**Fig. 1:**
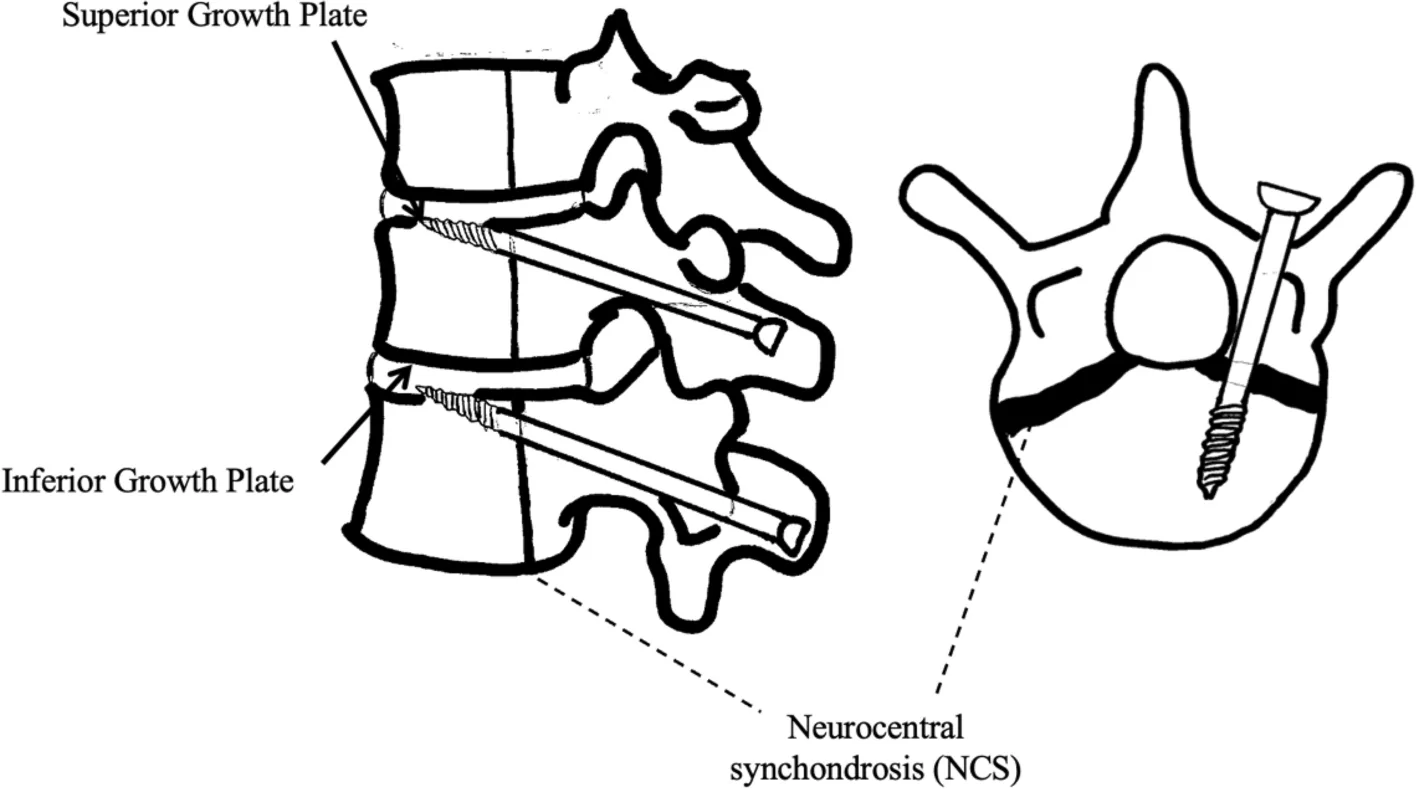
Schematic demonstrating intended screw trajectory

Four male pigs underwent posterior spinal instrumentation at eight weeks of age. Anesthesia was administered in accordance with IACUC-approved protocols previously described in porcine models [11, 22, 23]. After achieving adequate anesthesia, a paraspinal “Wiltsie” posterior approach was employed under sterile technique minimizing exposure of the posterior elements to avoid inducing auto fusion. An incision was made 2 to 3 cm lateral to the midline, and sharp dissection was carried down through the skin and subcutaneous tissue. Muscles were bluntly dissected after sharp incision of the fascia. The laminae were not exposed. Bony landmarks were palpated through the muscles and pedicle screw start points were identified using palpation and fluoroscopy. Four instrumentation levels (T9 to T12) were identified and confirmed with fluoroscopy for each animal. Unilateral instrumentation was performed in all specimens; the control group received pedicle screws (on the right side in one animal and left side in the other) and the experimental group received SET screws (on the right side in one animal and on the left in the other) (Fig. 1). A 0.045-inch Kirschner wire was used to localize the entry point at the base of the superior articular process (SAP). After fluoroscopic confirmation, an awl was advanced through either the pedicle (control group) or cranially directed to advance through the vertebral body, into the vertebral epiphysis, resting in the superior endplate (SET group) and followed with a guide wire. After confirmation of appropriate screw trajectory and length, a partially threaded, cannulated stainless-steel screw (4.0 mm diameter; length range 22–28 mm; OrthoPediatrics Corp., Warsaw, IN) was placed unilaterally. We chose the screw length and insertion depth in an attempt to traverse the superior end plate, but have minimal, if any, penetration into the disc space itself. Our goal was to cause growth inhibition on the superior end plate, but not disrupt the disc space. Screw length was measured using a ball-tip probe manually, and depth of insertion was monitored using lateral fluoroscopy.

The trans-physeal SET screw had a slightly more inferior starting point than a standard pedicle screw, aiming to reduce the likelihood of impingement during thoracic facet joint motion. Although it is intended to traverse the cranial endplate of the instrumented vertebra, the length was chosen to minimize penetration into the intervertebral disc above, intending to allow growth modulation without altering spinal motion and potentially preserving intervertebral disc health.

Following surgery, anesthesia was discontinued, and animals were provided routine postoperative care. The animals grew for three months postoperatively then were euthanized per IACUC protocol. Thoracic spines were harvested en bloc, including motion segments from the mid-thoracic spine through at least two lumbar levels.

### Imaging

All animals underwent dorsoventral and lateral X-rays before surgery, immediately following surgery, three months postoperatively, and after harvest. CT imaging was performed on all spines following harvest (slice thickness of 0.625 mm, General Electric LightSpeed Volumetric Computed Tomography 64-slice scanner, GE Healthcare, Chicago, IL). CT scans were acquired in instrumented spines, and repeated following implant removal.

Subsequently, thoracic spines were scanned using a Bruker 7.0 Tesla MRI scanner with a quadrature volumetric Transmit/Receive (T/R) coil. Each sample was scanned in two parts: the upper segments followed by the lower segments. An axial T1-weighted three-dimensional Fast Low Angle Shot (FLASH) sequence with isotropic voxels (TE = 8 ms, TR = 50 ms, matrix = 140 × 90 × 273, FoV = 42 mm × 27 mm × 80 mm, time = 22:07) and a coronal T2 Multi-Echo Multi Slice (MSME) sequence (TE = 9 ms, TR = 1666 ms, matrix = 336 × 192 × 10, FoV = 84 mm × 48 mm × 20 mm, # echoes = 14, time = 10:40) were acquired. From the T2 map sequence, the T2 relaxation time for each voxel was calculated by fitting the magnitude of the multi-echo data to a mono-exponential decay model (Si(TE) = S0 * e^(-TE/T2) + c), where *c* represents the Rician noise floor [23].

### Radiographic outcomes

**Fig.2:**
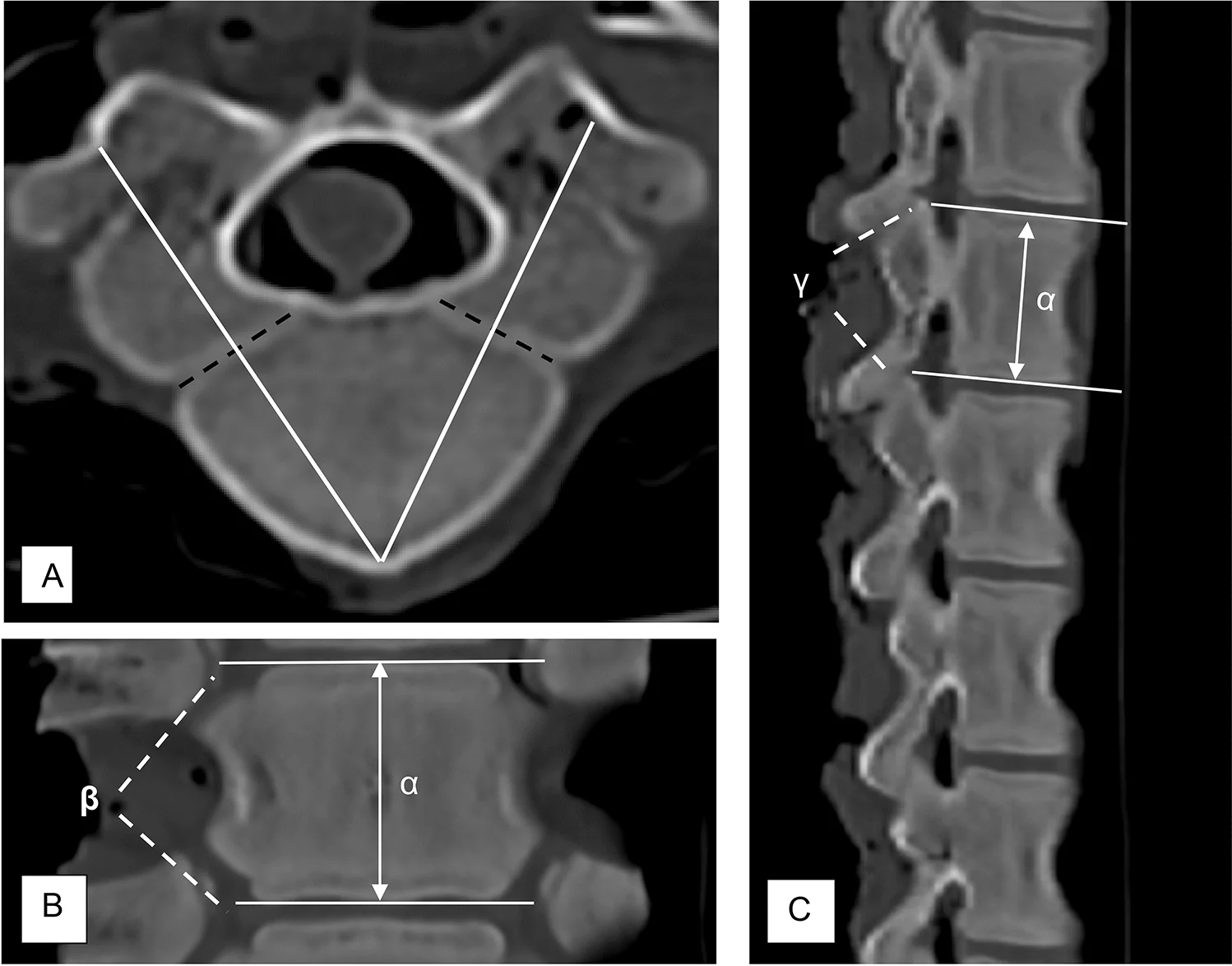
**A-C** **A** Pedicle length measurement (solid white line), bisecting midpoint of the neurocentral synchondrosis (dotted line). **B** Vertebral body height (α) and wedging (β) measurement on coronal view. **C** Vertebral body height (α) and wedging (γ) measurement on sagittal view

Coronal and sagittal curve magnitudes of the instrumented thoracic segments were measured from radiographs preoperatively and three months postoperatively using the standard Cobb angle technique. Preoperative and postoperative coronal and sagittal vertebral body wedging of the instrumented segments were measured. In addition, vertebral body heights (measured on the dorsoventral radiograph from the middle of the vertebra) of instrumented and un-instrumented segments (two levels above and below instrumented levels) were measured (Fig. 2). CT imaging was evaluated for the presence of inadvertent fusion masses, physeal bars, and premature physeal closure, and evidence of facet joint degeneration was classified according to the Pfirrmann classification [26]. MR images were reviewed for signs of disc degeneration, as well as for disc morphology and water content, following a previously published protocol validated in a porcine model [22, 26] and classified per the Fujiwara classification [27].

Pedicle length was measured on axial CT scans in both instrumented and un-instrumented spinal segments to determine whether screw threads traversing the neurocentral synchondrosis (NCS) had any effect on pedicle growth. For each pedicle, the NCS was identified and used as a reference for measurement. Pedicle length was defined as the distance from the anterior aspect of the vertebral body, passing through the midpoint of the NCS, to the posterior cortex (Fig. 2).

### Statistical analysis

Analysis of variance (ANOVA) was used to evaluate changes in radiographic measures over time between experimental and control groups and individual vertebral levels. Multivariate analysis of variance (MANOVA) was used to assess between-group differences in the radiographic measures, with the presence of SET screws versus pedicle screws as the independent variable. The alpha level for significance was set at 0.05. Categorical dependent variables were analyzed for proportional differences between the two study groups using cross-tabulation and the chi-square statistic (*p* < 0.05).

## Results

**Fig. 3:**
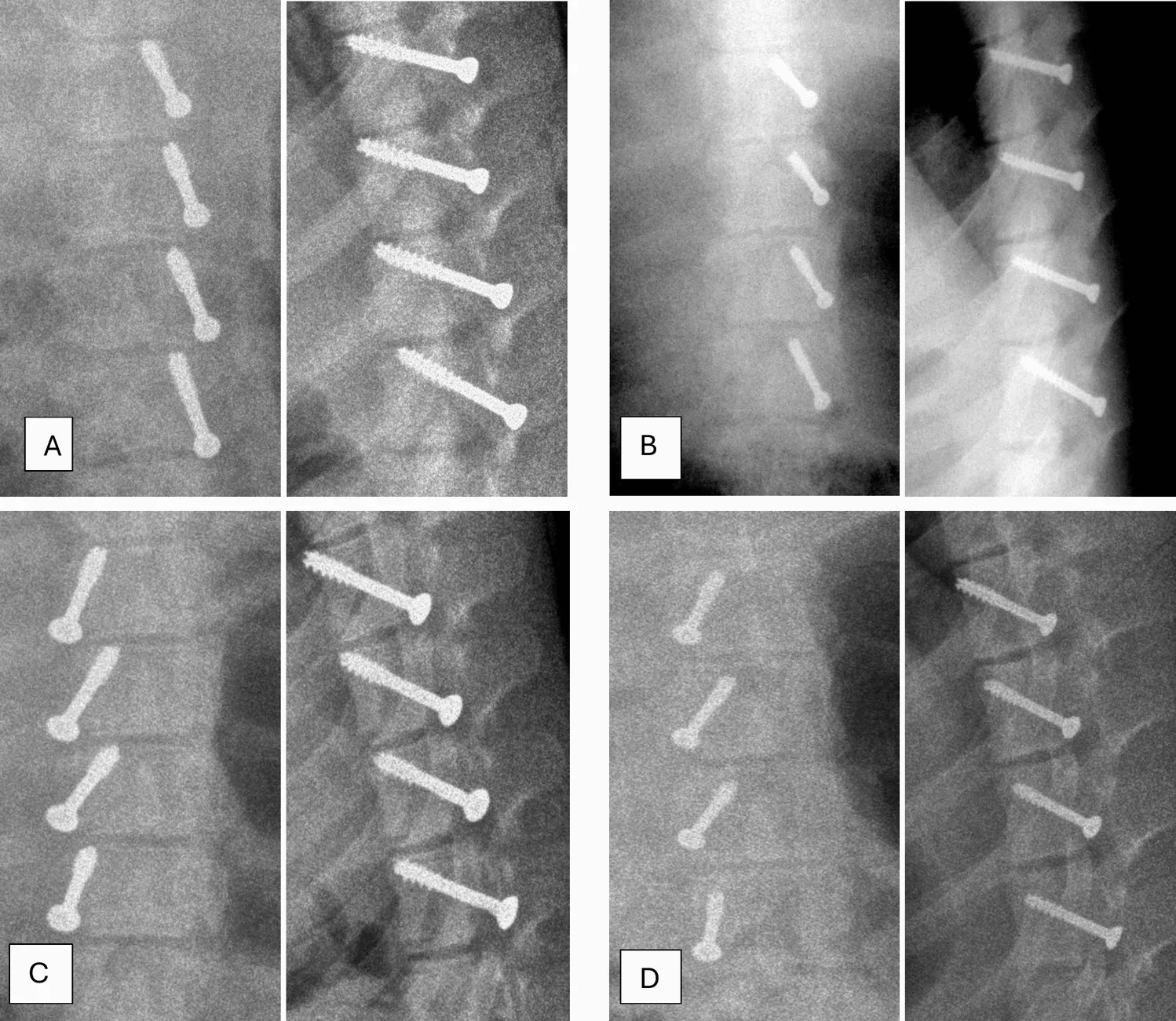
**A-D** Anteroposterior and lateral radiographs demonstrating spinal epiphysiodesis trajectory (SET) screw placement at levels T9 to T12. Case 1: **A** immediately postoperatively and **B** at 3-month follow-up. Case 2: **C** immediately postoperatively and **D** at 3-month follow-up

**Fig. 4:**
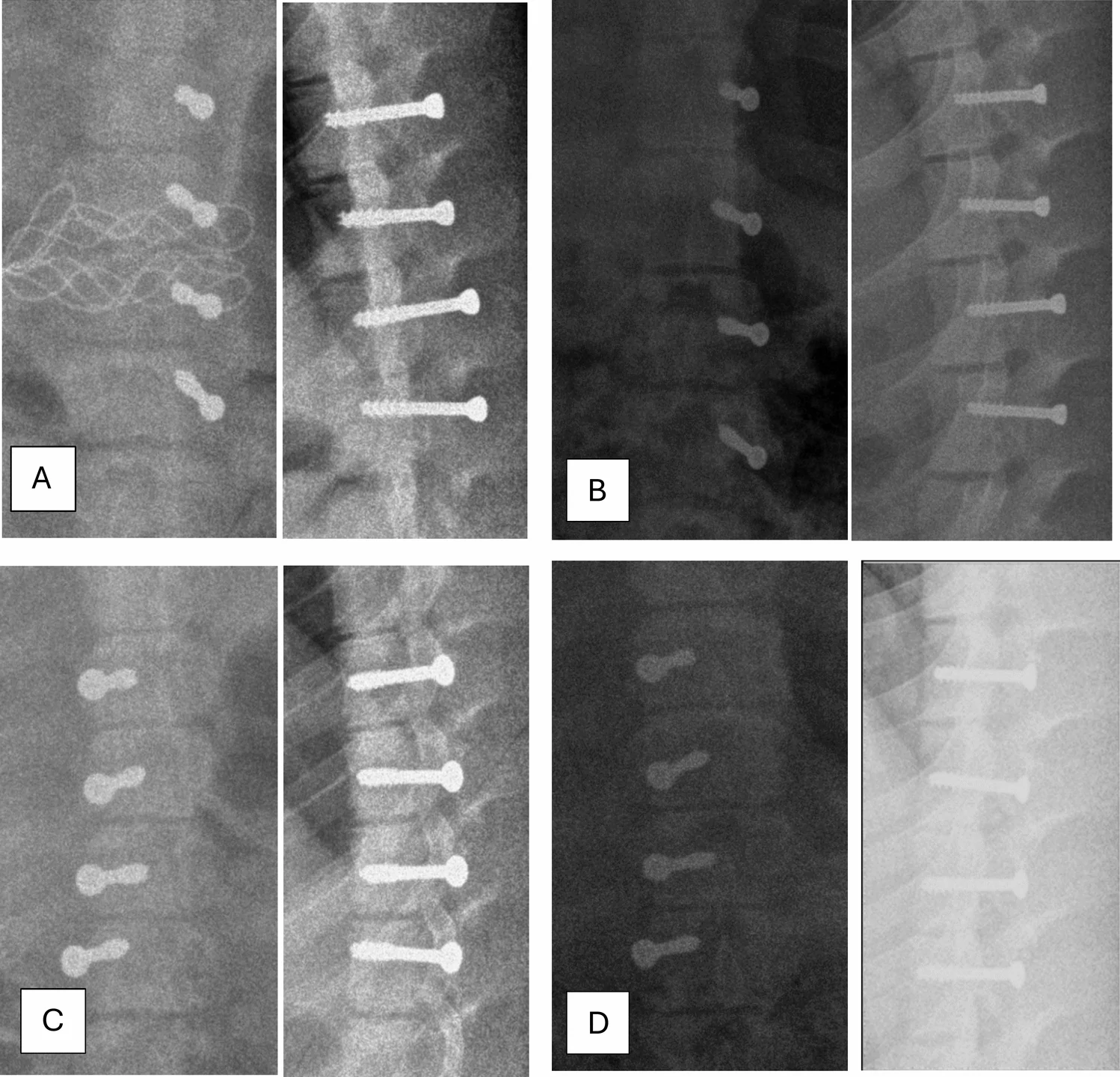
**A-D** Anteroposterior and lateral radiographs demonstrating pedicle screw instrumentation at levels T9 to T12. Case 1:**A** immediately postoperatively and**B** at 3-month follow-up. Case 2:**C** immediately postoperatively and**D** at 3-month follow-up

Four male piglets (two in pedicle screw group, two in SET screw group), aged a mean 8.5 ± 0.1 weeks at the time of surgery, underwent posterior spinal instrumentation (Figs. 3, 4). Over the 12-week growth period, mean weight increased from 14.9 ± 1.8 kg to 36.4 ± 4.6 kg, and mean body length increased from 61.5 ± 3.9 cm to 84.5 ± 5.1 cm.

**Fig. 5:**
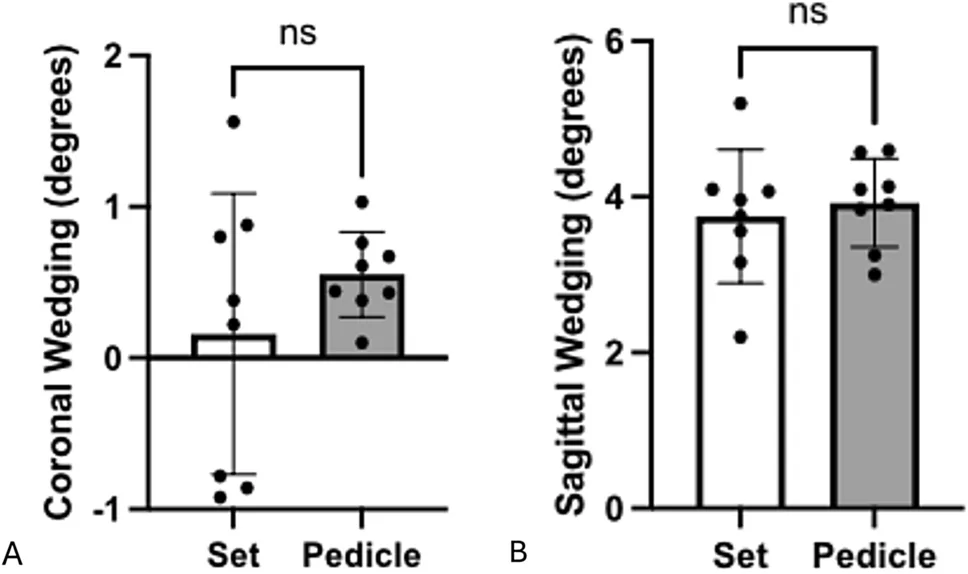
**A-B** Differences between spinal epiphysiodesis trajectory (SET) and pedicle screw groups in**A** coronal and**B** sagittal vertebral wedging. *ns = *p *> 0.05

**Fig. 6:**
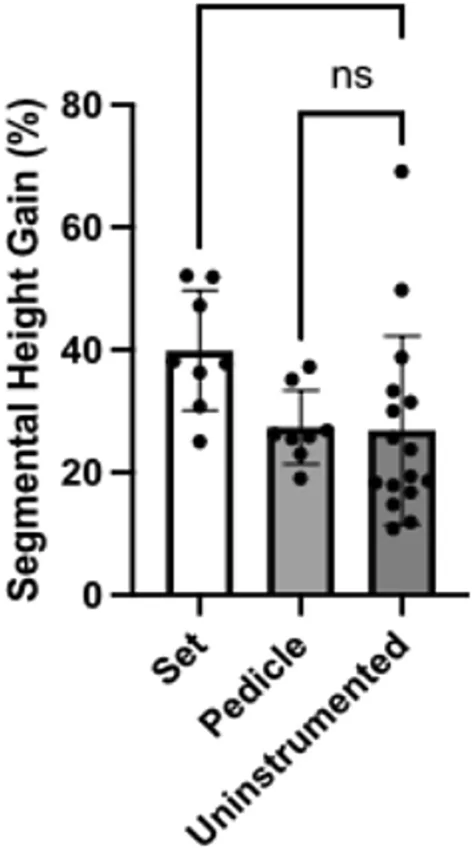
Differences in segmental vertebral height gain between spinal epiphysiodesis trajectory (SET) screws, pedicle screws, and un-instrumented groups. *ns:*p* > 0.05

Radiographic imaging revealed no significant differences in coronal or sagittal curve magnitudes between the SET and pedicle screw groups at both preoperative and postoperative assessments (*p* > 0.05). Additionally, no changes greater than 5° in curve magnitude were observed in either group (Table 1). Neither screw type induced coronal or sagittal vertebral wedging, and no significant differences were identified between SET-instrumented, pedicle-instrumented, and un-instrumented vertebrae for wedging (*p* > 0.05) (Fig. 5A-B). Neither SET nor pedicle screws limited vertical growth of the instrumented vertebrae. The mean vertebral height gain of the SET screw group was significantly greater than that of the pedicle screw group (*p* = 0.008) and the un-instrumented group (*p* = 0.042) (Fig. 6).

**Table 1:** Mean Preoperative and 3 Month Postoperative Sagittal and Coronal Cobb Angles in Pedicle and Spinal Epiphysiodesis Trajectory (SET) Screw groups

	Pedicle (*n* = 2)	SET (*n *= 2)	Effect Size (g)	*p*-value
Preoperative Sagittal (degrees ± SD)	13.28 ± 0.62	13.42 ± 1.04	–0.10	0.88
Postoperative Sagittal	10.28 ± 0.98	12.6 ± 0.62	–1.63	0.13
Preoperative Coronal	3.70 ± 0.23	1.19 ± 1.17	1.70	0.19
Postoperative Coronal	2.85 ± 0.13	–0.40 ± 3.04	0.86	0.37
Δ Sagittal	–3.00 ± 1.60	–0.82 ± 0.42	–1.07	0.29
Δ Coronal	–0.86 ± 0.36	–1.59 ± 1.87	0.46	0.67

**Fig. 7:**
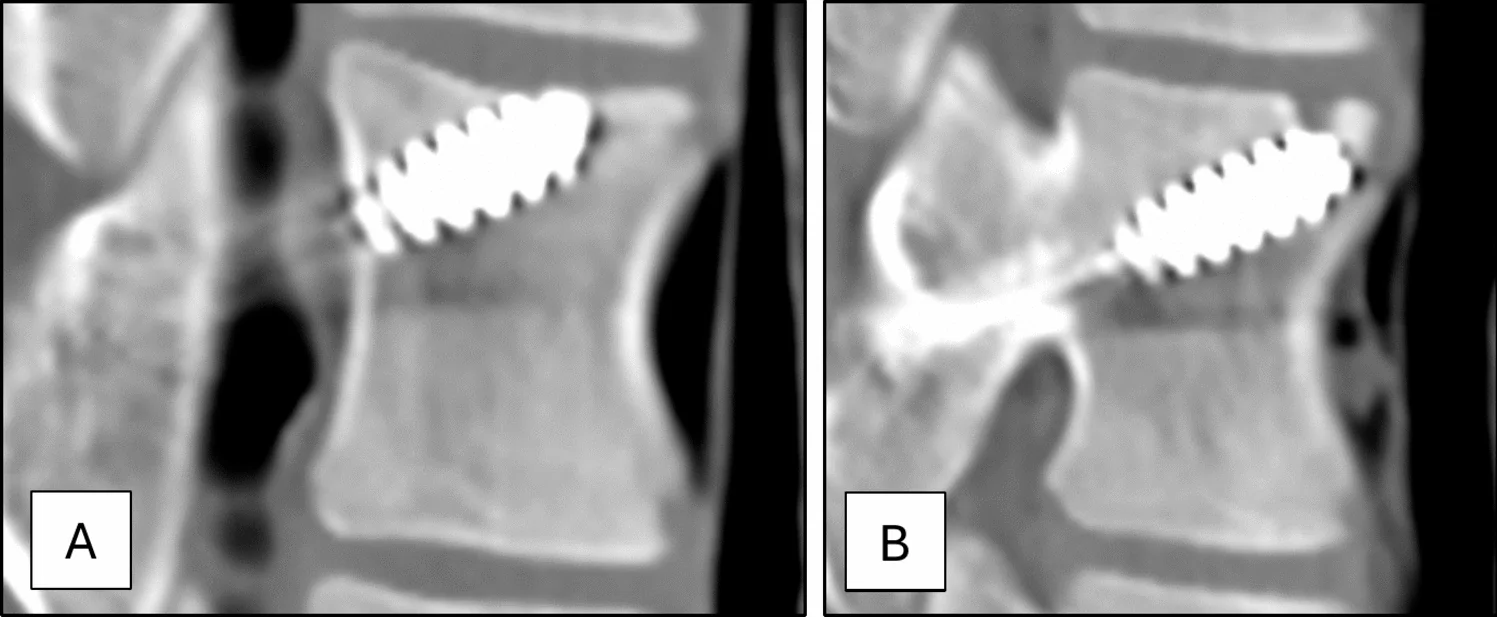
**A,B** Computed tomography (CT) images of vertebrae with spinal epiphysiodesis trajectory (SET) screws.**A** Vertebra with a SET screw that remained within the epiphysis at the end of the study.**B** Vertebra that “grew off” the SET screw, meaning the screw no longer crossed the epiphysis at study completion—a phenomenon previously described in screw-mediated growth modulation. The cavitation at the epiphyseal region in**B** suggests that the screw initially traversed the epiphysis at the time of implantation

**Fig. 8:**
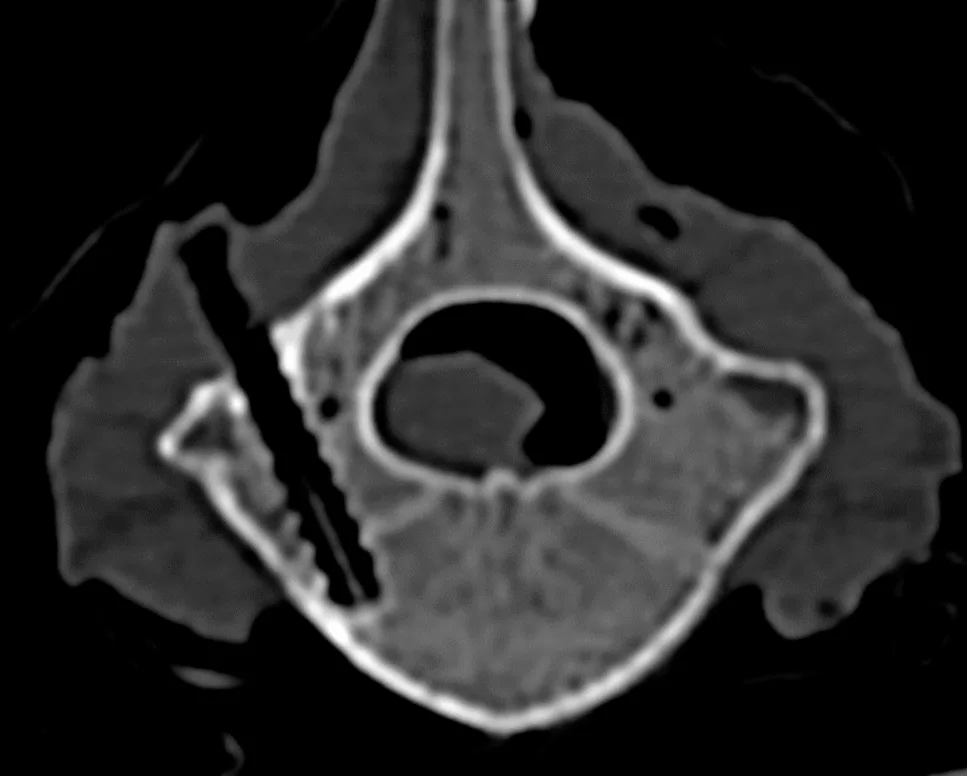
Computed tomography (CT) scan showing the threaded portion of a partially threaded spinal epiphysiodesis trajectory (SET) screw inadvertently traversing the neurocentral synchondrosis (NCS)

Among the SET screw group, immediate postoperative radiographs demonstrated appropriate seating of the screw within the epiphysis in all eight instrumented vertebrae. However, CT imaging three months later revealed that the screw track crossed the vertebral endplate in only six of eight vertebrae across the two animals, suggesting that successful instrumentation of the epiphysis occurred in only six vertebrae despite its radiographic appearance immediately postoperatively. Furthermore, CT imaging revealed that the vertebral endplate appeared to have “grown off” of the screw in five out of six vertebrae as evidenced by the final screw position being in the vertebral body but a screw track evident in the endplate. At the end of the experiment, only one of the eight SET screws remained positioned in the endplate (Fig. 7). In both the pedicle and SET screw groups, all screws crossed the neurocentral synchondrosis (NCS), primarily with the threaded portion of the screw, while the non-threaded portion of the screw traversed the NCS in only one of the SET screw vertebrae (Fig. 8). In order to evaluate if instrumentation across the NCS affected growth, we compared pedicle length between instrumented and un-instrumented pedicles with paired t-tests and found no significant differences in pedicle length between un-instrumented pedicles and those instrumented with SET or pedicle screws.

No significant changes in disc or facet joint health were observed based on the Pfirrmann [26] and Fujiwara [28] classifications. No physeal bars were detected, although one SET screw specimen exhibited minor endplate irregularities.

## Discussion

In this pilot study conducted in a porcine model, SET screws did not induce scoliotic or kyphotic deformity. Given that other methods, such as screw epiphysiodesis of the NCS [21, 29] and endplate ablation epiphysiodesis using an electrical current [30], have been shown to result in spinal deformity, further investigation is warranted to define the mechanisms and timing of spinal growth, which are essential for facilitating spinal growth modulation and deformity correction.

In an immature porcine model, Zhang and Sucato demonstrated that pedicle screws alone can induce and reverse a scoliotic deformity. The mechanism of deformity creation was thought to be asymmetric epiphysiodesis of the NCS [22, 29]. In this study, unilateral transpedicular screw fixation traversing the NCS produced asymmetric growth of the synchondrosis, generating scoliosis with convexity on the side of the screw placement [22]. A group of pigs with experimentally induced scoliosis was later treated with placement of a second set of double pedicle screws in the contralateral pedicles, which resulted in partial correction of the deformity [29]. However, this method also has drawbacks, such as a reduction in spinal canal area and depth, highlighting the need for further research into alternative growth modulation techniques [31].

In a rabbit model, Dodge, Bowen, and Jeong demonstrated that the application of electrical currents at an amplitude sufficient to induce hemi-epiphysiodesis inhibited growth on one side of the vertebral endplate [32]. Their findings showed that this induced hemi-epiphysiodesis created an in vivo model of scoliosis in which the concavity on the scoliosis created is on side of hemi-epiphysiodesis. The authors suggested that this technique could potentially promote asymmetrical spinal growth and correct scoliotic curves.

In humans, longitudinal spinal growth primarily occurs through ossification of the superior and inferior vertebral body cartilaginous endplates adjacent to the discs [33, 34]. Given that eccentric placement of trans-physeal screws has proven effective for epiphysiodesis with favorable outcomes in the correction of angular deformities across various settings [2–4], we investigated SET screws for spinal growth modulation. The potential clinical applications of SET screws are broad. For example, SET screw-mediated spinal deformity correction could be developed as a motion-preserving treatment for scoliosis in growing children, either alone or as a supplement to bracing. Additionally, posterior spinal fusion using SET screws could be explored as a technique to prevent crankshaft during definitive spinal fusion in an immature skeleton. However, the growth modulation potential of SET screws was not demonstrated in our study, and several methodological and biological factors may have contributed to this negative finding.

In some of our cases, intraoperative evidence suggested that the screw had penetrated the upper endplate. However, CT imaging after the growth period revealed that the screw was no longer positioned in the epiphysis, indicating that the vertebra may have “grown off” of the screw, a phenomenon previously described in screw-mediated growth modulation [34, 35]. The “growing off” phenomenon has been observed in other examples of screw-mediated growth modulation, most notably in attempted growth modulation of the proximal femoral epiphysis. Screws introduced in an antegrade fashion from the metaphysis into the epiphysis can become anchored more firmly in the metaphysis; as growth proceeds, the threaded portion of the screw may no longer traverse the physis, thereby losing its ability to directly modulate growth. Studies of the proximal femoral epiphysis have demonstrated that a more eccentric placement of the screw increases both the risk of growing off and the efficacy of correction [36]. This growing-off phenomenon may have limited the ability of the screws to modulate growth in the model as tested. Strategies to mitigate this effect could include deeper penetration of the screw into the epiphysis and disc space, although this may also increase the likelihood that the technique, as described, would induce degenerative disc disease. Another strategy might be to use screws with larger diameter and shorter threaded regions, increased pull-out strength at the tip of the screw where it penetrates the epiphysis, but not in the metaphysis. We suspect that extension of the screw across the disc space and into the inferior endplate of the vertebra above would be far more likely to induce growth modulation. However, we also suspect that this method would be very likely to induce degenerative disc disease due to its limitation of spinal mobility. For this reason, we elected not to test this technique in the present study. The bilateral placement of SET screws across the disc space and into the inferior endplate of the vertebra above may, however, have potential as a method for anterior growth arrest in the setting of definitive spinal fusion in an immature patient; however, we feel that trans-discal screws would likely not be a useful strategy when the intent is to correct deformity while preserving spinal motion. In cases where the screw remained positioned in the endplate, only a small portion of the cross-sectional area of the endplate was traversed by the screw, which may have been insufficient to effectively modulate growth. Additionally, we made a conscious effort to place the SET screws within the epiphysis while minimizing, or avoiding, penetration of the intervertebral disc. This approach resulted in very few threads crossing the physis, which may have further limited the growth modulation potential of the screws. Our approach was only designed to affect the superior endplate of each vertebra, which may not have been powerful enough to induce deformity. Little is known about the relative contributions of the superior and inferior endplates to longitudinal growth.

Furthermore, for both the pedicle and SET screw groups, we used short, partially threaded screws in an attempt to place only the threaded portion of the screws past the NCS. The goal was to leave a smooth portion of the screw across the NCS to avoid growth arrest, as it has been described previously that fixation across the NCS induces a scoliotic deformity particularly in immature animal models [22, 29, 31]. Despite this intention, the threads crossed the NCS in most of the instrumented vertebra. However, we observed no deformity in either the pedicle or SET screw groups, in disagreement with previous research. We also found no difference in pedicle length associated with instrumentation across the NCS. It is possible that the lack of deformity in this study was due to insufficient remaining growth in the NCS to induce a deformity after its arrest.

This study has several limitations. First, porcine spine anatomy differs from human spine anatomy due to the presence of an epiphyseal bone. As a SET screw would have additional fixation in a bony epiphysis, we felt this would increase the likelihood that it would cause growth modulation in our animal model but potentially make this result less transferable to humans. Since we failed to demonstrate growth modulation using our method in a porcine model, we surmise that this makes the method even less likely to succeed in humans. Additionally, there are differences in spinal biomechanics that may affect spinal growth and development when using a quadruped animal model. Although the porcine model is well established in spinal research, these factors may affect the translatability of the results. The evaluation of results in this study was conducted after only three months of growth, whereas in clinical practice, implants would typically remain in place for several months or years until the deformity is corrected or skeletal maturity is reached. The postoperative period used may have been too short to detect signs of iatrogenic degenerative disease. The sample size in this pilot study is another clear limitation; however, since little change was found in this small pilot, additional animals using the same study protocol are not likely warranted. As such, it is not yet possible to draw definitive conclusions about how this technique might modulate spinal growth or correct spinal deformities in humans.

## Conclusion

This pilot study explored a novel approach to spinal growth modulation using SET screws. Although no significant deformity was observed, these negative results provide a foundation for future research into vertebral growth mechanisms and the potential for modulating spinal deformity. Further studies are needed to clarify how endplate disruption influences growth. It remains to be seen whether variations on the SET screw concept may offer potential for spinal growth modulation.

## Data Availability

The data that support the findings of this study are available from the corresponding author upon reasonable request.
